# Gravity Affects the Closure of the Traps in *Dionaea muscipula*

**DOI:** 10.1155/2014/964203

**Published:** 2014-07-15

**Authors:** Camilla Pandolfi, Elisa Masi, Boris Voigt, Sergio Mugnai, Dieter Volkmann, Stefano Mancuso

**Affiliations:** ^1^DISPAA, University of Florence, Viale delle idee 30, 50019 Sesto Fiorentino, Italy; ^2^IZMB, University of Bonn, Kirschallee 1, 53115 Bonn, Germany

## Abstract

Venus flytrap (*Dionaea muscipula* Ellis) is a carnivorous plant known for its ability to capture insects thanks to the fast snapping of its traps. This fast movement has been long studied and it is triggered by the mechanical stimulation of hairs, located in the middle of the leaves. Here we present detailed experiments on the effect of microgravity on trap closure recorded for the first time during a parabolic flight campaign. Our results suggest that gravity has an impact on trap responsiveness and on the kinetics of trap closure. The possible role of the alterations of membrane permeability induced by microgravity on trap movement is discussed. Finally we show how the Venus flytrap could be an easy and effective model plant to perform studies on ion channels and aquaporin activities, as well as on electrical activity *in vivo* on board of parabolic flights and large diameter centrifuges.

## 1. Introduction

The response of Venus flytrap (*Dionaea muscipula*) to mechanical stimulation has long been known, and it is one of the most rapid movements in the plant kingdom [1, 2].

The plant produces a rosette of leaves, each divided into two parts: a lower part called the lamina and the upper part called the trap. The trap catches prey thanks to a very rapid movement of its bilobed halves that shut when the trigger hairs are stimulated. At room temperature, two touches activate the trap, which snaps shut in a fraction of second [3]. At higher temperature only one stimulus is required for trap closure [4]. The stimulation of the trigger hairs activates mechanosensitive ion channels and generates receptor potentials, inducing the action potentials (APs) that initiate the closure [5]; electrical signals are the immediate cause of the trap movements, irrespective of the way in which the signal is triggered (mechanical stimulation or electrostimulation) [5]. Once the insect is caught, the lobes seal tightly allowing digestion to take place [6, 7]. The APs in* Dionaea muscipula* have been extensively studied (e.g., [5, 8, 9]). Trigger hair-induced generation of action potentials is not exclusively associated with the trap closure. The struggling of the entrapped prey in the closed trap results in the generation of further action potentials which cease to occur just when the prey stops moving. These APs may induce inhibition of the dark reaction of photosynthesis [10] showing that chlorophyll-A fluorescence is under electrochemical control [11]. Although this spectacular example of plant movement has long fascinated scientists, the mechanism by which the trap works remains poorly understood [12]. Some explanations proposed involve an irreversible cell wall loosening, induced by the acidification of the cells [6], or a rapid loss of turgor pressure similarly to what happens in stomata [13]. However, the validity of both mechanisms has been questioned because they cannot explain the speed at which the movement happens. More recently, other two models have been proposed: the elastic deformation that results from a snap-buckling instability [14] and a hydroelastic curvature mechanism based on the fast opening of water channels [9]. Both models may convincingly account for the speed of the movement.

The possibility to study the effect of microgravity on living organisms is a unique opportunity to observe the alteration of phenomena in the absence of the otherwise omnipresent gravity force. Although the understanding of the effect of gravity on animal and plant bodies is crucial, in view of the possible future space travels, the research is moving slowly if compared with other research fields, due to the accessibility to microgravity conditions and the challenging experimental condition.

In the present study we report the effect of microgravity on trap closure conducted during a parabolic flight campaign. This gave us the opportunity to test* Dionaea muscipula* as possible candidate to study the effect of gravity on the electrical activity of organisms by monitoring the variation in the excitability of the traps and the alteration in the kinetics of their closure. Furthermore, the changes in the kinetics of trap closure gave us important hints on the mechanism at the base of the fast trap snapping.

## 2. Materials and Methods

Parabolic flight experiments were performed in an A300 airplane (Novespace, France) during the 9th DLR parabolic flight campaign. A typical parabolic flight manoeuvre provides alternating acceleration levels of regular gravity (1 g), microgravity (0 g) for 22 s, and two periods (20 s) of hypergravity (up to 1.8 g for 20 s) before and after each period of microgravity. Twenty* Dionaea muscipula* J. Ellis plants were grown in a growth chamber with 14/10 h light/dark period, in well-drained peat moss in plastic pots irrigated with distilled water. All experiments were performed on healthy adult specimens. The pots were sealed with parafilm to avoid the floating of the substrate during the zero gravity periods, and plants were secured inside a plexiglass growth chamber. Digital HD video recorder Sony HDR-SR11E was used to film the Venus flytraps at 25 fps. Each day experiments were done on 4 different plants. All the traps were mechanically stimulated during 0, 1, and 2 g, using a wooden stick by gently touching the trigger hairs. The wooden stick was immediately removed after stimulation. The collected movies were analysed frame by frame, and the trap closure was quantified by measuring the change of distance between two laminas with ImageJ software [15]. Space and time constraints limited the number of plants that could be carried on board and consequently limited the number of traps available each day. More emphasis was given to the zero gravity condition; therefore 25 traps were tested in microgravity, 20 were tested in 1 g during the flight, and 8 traps were devoted to 2 g. The distance *y*(*t*) between the edges of the trap leaf was measured in the closing process. In the open state, the distance between the edges of the trap leaf is *y*_max⁡_. As individual plants have different opening distances data were normalized *x* = *y*/*y*_max⁡_. The speed of trap closure was calculated as *v* = *dx*/*dt* and it has the dimension of s^−1^.

## 3. Results

**Figure 1:**
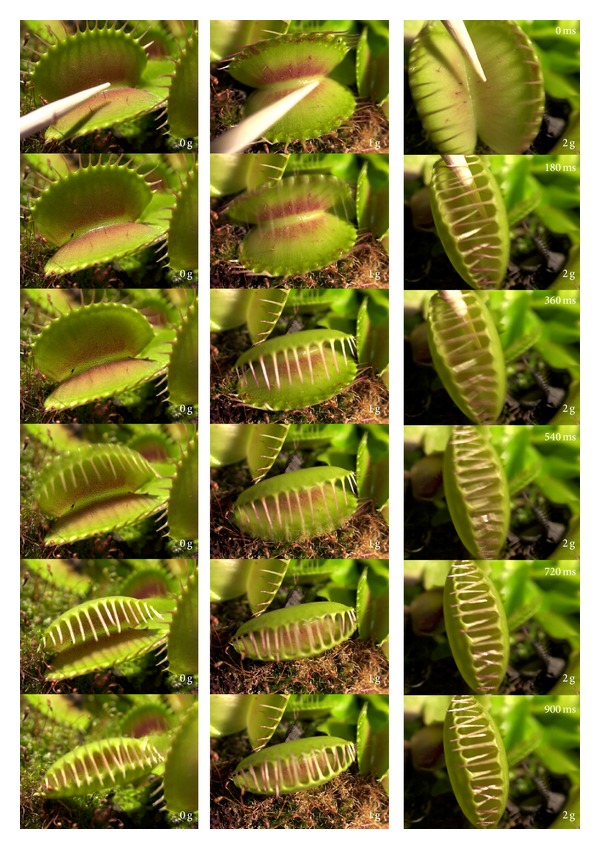
Closing of the trap in micro- (0 g), normal (1 g), and hypergravity (2 g).

The trap closure was studied at different gravity conditions (Figure 1).

Results showed a low responsiveness of traps in microgravity: 36% of the traps did not close at all and 48% manifested a slower closing motion. The traps stimulated in normal gravity demonstrated a normal closure in 80% of the cases and, finally, the traps stimulated in hypergravity reacted promptly to the stimulation, with 50% of the traps being faster than controls (Table 1).

**Figure 2:**
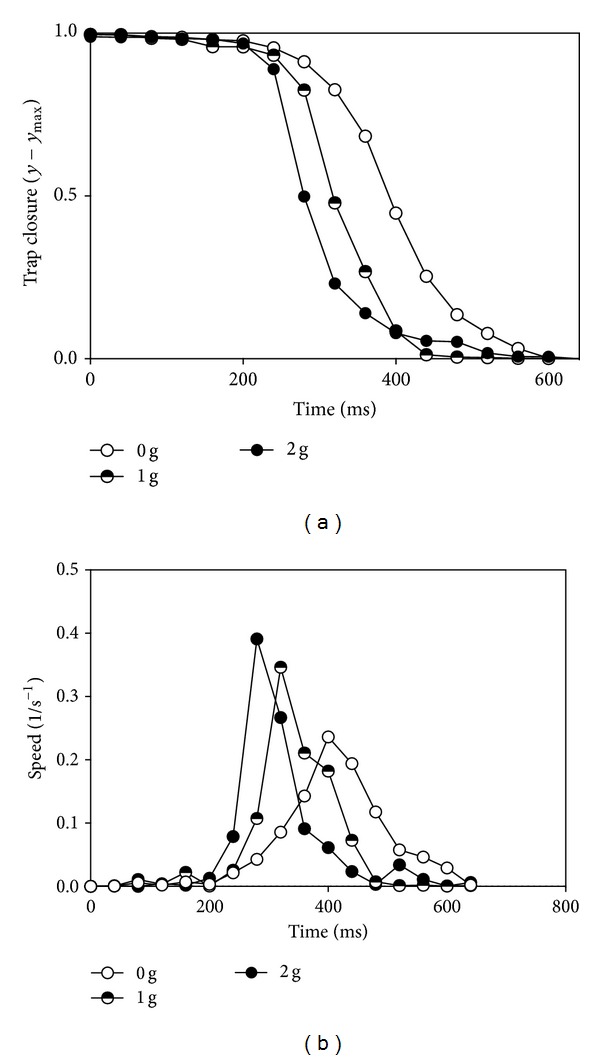
Effect of gravity on trap closure. (a) Kinetics of trap closure under different gravity conditions: *y* is the distance between the edges of the lobes. (b) Dependency of the speed of trap closure on time after stimulation.

In the graph (Figure 2) three representative examples have been reported.

Trap closure is strongly affected by gravity: in microgravity the kinetics of snapping is slower (Figure 2(a)) and the acceleration is low if compared with 1 and 2 g where the speed of closure increases sharply after the trigger (Figure 2(b)).

Because of the constraints involved in performing the experiments on a plane we were unable to measure the reaction time between the trigger and the start of the closure (our time resolution was 40 ms and the traps were stimulated manually). However, visual observation revealed a delayed response in zero gravity, and an anticipated response in hyper-g.

## 4. Discussion

Volkov et al. described the trap closure as consisting of three different phases [9]: (1) a mechanically silent period with no observable movement immediately after stimulation; (2) the period when the movement starts accelerating; (3) the fast movement of the trap, when the leaves quickly relax to the new equilibrium state. In our results it appears that microgravity acts in* Dionaea* at two different levels: (i) by impairing the signal transduction, as suggested from the high percentage of inactive traps and from the lower responsiveness (phase 1); and (ii) by altering the trap kinetics by significantly reducing the trap closing time (phases 2-3), eventually suggesting that the mechanisms leading to trap closure are gravity-related. The electrical properties of excitable cells are extremely important in higher organisms. Changes of their parameters under microgravity can impair the functionality of the neural systems and have significant consequences for human, especially in view of long space travels. The few reports available on animal cells suggest that action potentials are affected by gravity [16]: in particular, the propagation velocity and their intensity seem to be gravity-dependent; that is, they increase under hypergravity and decrease under microgravity compared to 1 g [17]. Very little is known for higher plants. Masi et al. monitored for the first time the electrical activity of root cells during a parabolic flight and observed alterations of the frequency of APs [18]. Altered parameters have been reported also under hypergravity (Masi et al., unpublished) suggesting that the excitability of both plant and animal cells is heavily affected by altered gravity conditions.

In* Dionaea muscipula* it is well known that the stimulation of trigger hairs generates the two APs required for trap closure [9, 19, 20]. The high number of inactive traps (i.e., no response to the trigger) and the apparent slower response time of the trap closure suggest an alteration in the generation or propagation of the APs in microgravity. In plants, as well as in animals, action potentials are induced by the fast opening and closing of ion channels whose functionality has been little studied and understood in microgravity so far. In fact, ion channels are integral membrane proteins, and they could be affected either directly or indirectly by gravity. Gravity could directly affect the protein integrity, whereas changes in the thermodynamical properties of the membrane could have an indirect effect on the ion channel functionality [21]. In 2001, Goldermann and Hanke showed for the first time that gravity influences the integral open state probability of ion channels providing a first explanation of the effects of gravity on electrical signalling [22]. Those findings were further confirmed by patch-clamp analysis [21].

Nothing similar has been done for plants. The first silent stage of the trap closing involves transduction of electrical signal and hence it is related to ion channel gating. Interestingly results similar to the ones obtained here under microgravity were observed when applying channel blockers to the traps [9]. The use of BaCl2, ZnCl2, and TEACl significantly delayed trap closure and altered its speed [9].

Of course, the results presented here are just preliminary. Further studies will be necessary to consolidate the results and to investigate in deeper detail the possible effect of gravity on the generation and propagation of action potentials. Particularly interesting would be to stimulate electrically the traps allowing measuring and quantifying the delay in trap closure under altered gravity conditions.

To conclude, our results demonstrate the role of microgravity on the events leading to trap closure. The possible alterations of ion and water channel permeability that could be at the base of the lower responsiveness and slow closure observed in microgravity are a possibility worthy to be investigated. In fact, if properly demonstrated it would strengthen the validity of the hydroelastic curvature model suggested by Volkov et al. [9]. Finally, we want to stress the fact that Venus flytrap could be an easy and effective model plant to perform studies on ion channels and aquaporin activities, as well as on electrical activity* in vivo* on board of parabolic flights and large diameter centrifuges.

## Figures and Tables

**Table 1:** Trap behaviour recorded under different gravity conditions; the number of traps tested *n* is reported in the table.

	*n*	No response	Normal closure	Slow closure	Fast closure
0 G	25	36%	16%	48%	0%
1 G	20	10%	80%	10%	0%
2 G	8	0%	50%	0%	50%
